# North American *(Panax quinquefolius)* and Asian Ginseng *(Panax ginseng)* Preparations for Prevention of the Common Cold in Healthy Adults: A Systematic Review

**DOI:** 10.1093/ecam/nep068

**Published:** 2011-02-14

**Authors:** Jennifer Krebs Seida, Tamara Durec, Stefan Kuhle

**Affiliations:** ^1^School of Public Health, University of Alberta, 650 University Terrace, Edmonton, Alberta, Canada T6G 2T4; ^2^Durec Information Services Inc., St Albert, Alberta, Canada

## Abstract

*Background*: Standardized ginseng extract has become the best-selling cold and flu remedy in Canada, yet much controversy regarding the efficacy of ginseng in preventing common colds remains. *Objective*: To assess the efficacy of ginseng preparations for the prevention of common colds in healthy adults. *Methods*: Comprehensive bibliographic database, trial registry and grey literature searches were conducted up to December 2007. Randomized controlled trials or controlled clinical trials comparing North American (*Panax quinquefolius*) or Asian ginseng (*Panax ginseng*) root extract to placebo or no treatment in healthy adults were included. Two reviewers independently applied the study selection criteria and assessed methodological quality. *Results*: Five trials involving 747 participants were included. All five trials examined North American ginseng. The methodological quality of the trials varied widely. Ginseng preparations significantly reduced the total number of common colds by 25% compared to placebo (one trial; 95% CI: 5–45). There was a tendency toward a lower incidence of having at least one common cold or other acute respiratory infection (ARI) in the ginseng group compared to the placebo group (five trials; relative risk: 0.70; 95% CI: 0.48–1.02). Compared to placebo, ginseng significantly shortened the duration of colds or ARIs by 6.2 days (two trials; 95% CI: 3.4–9.0). *Conclusions*: There is insufficient evidence to conclude that ginseng reduces the incidence or severity of common colds. North American ginseng appears to be effective in shortening the duration of colds or ARIs in healthy adults when taken preventatively for durations of 8–16 weeks.

## 1. Introduction

Common colds are mild upper respiratory tract infections that are characterized by coughing, nasal stuffiness and discharge, sneezing and sore throat. The common cold is one of the most prevalent and widespread sources of morbidity throughout the world, and adults experience an average of two to four colds annually. Although the common cold is generally a mild, self-limiting condition, it is associated with a significant economic burden due to medical costs and lost work time [1].

Common colds are one of the most frequent conditions for which natural health products are used [2]. One such alternative remedy that has gained much popularity in Canada during recent years is COLD-fX, a proprietary natural supplement isolated from North American ginseng root (*Panax quinquefolius*). This ginseng preparation is heralded as the “uncontested Canadian leader in the prevention of colds” and has gained widespread use, such that it now ranks as the country's best-selling cold and flu remedy [3]. However, much debate regarding the efficacy of COLD-fX in the prevention of common colds continues.

Recently, several clinical trials have investigated the efficacy of COLD-fX in reducing the incidence of common colds in adults. The purpose of this review is to describe and assess the evidence on the efficacy of COLD-fX and other North American or Asian ginseng root extracts in reducing the number, severity and duration of common colds and cold symptoms in healthy adults.

## 2. Methods

### 2.1. Search Strategy

The research librarian (T.D.), in collaboration with the researchers, developed and implemented search strategies designed to identify the highest level of evidence available. Search terms were adapted appropriately to search the following electronic databases from their inception to December 2007: Alt HealthWatch (1990 to present), AMED (1985 to present), CINAHL (1937 to present), Cochrane Central Register of Controlled Trials (3rd Quarter, 2007), Cochrane Database of Systematic Reviews (Issue 2, 2007), Database of Abstracts of Reviews of Effects (1994 to present), EMBASE (1988 to present), Evidence-based Complementary Medicine (2005 to present), International Pharmaceutical Abstracts (1970 to present), Natural Standard, OCLC PapersFirst and ProceedingsFirst (1993 to present), Ovid MEDLINE (1950 to present), ProQuest Dissertations and Theses (1861 to present), BIOSIS Previews (1969 to present), Science Citation Index Expanded (1900 to present) and Social Sciences Citation Index (1956 to present). The appendix outlines the MEDLINE search terms and strategy that were subsequently adapted to accommodate the controlled vocabulary and search language of each database.

Trial registers including ClinicalTrials.gov, Current Controlled Trials metaRegister, National Research Register and complementary and alternative medicine websites including HerbMed, NCAM (National Center for Complementary and Alternative Medicine) (http://nccam.nih.gov), NIH Office of Dietary Supplements (http://ods.od.nih.gov), CAMEOL (Complementary and Alternative Medicine Evidence OnLine) (http://www.rccm.org.uk/cameol/Default.aspx) and IBIDS (International Bibliographic Information on Dietary Supplements) (http://ods.od.nih.gov/Health_Information/BIDS.aspx) were searched for additional trials and unpublished literature.

### 2.2. Study Selection

Randomized controlled trials (RCT) and controlled clinical trials (CCT) were eligible for inclusion in this review. Participants in the primary studies were required to be adults (≥18 years) and be in good general health, as defined by the trial authors. Studies were considered for inclusion if participants in the treatment group received either: (i) COLD-fX, a proprietary standardized extract of North American ginseng root (CV Technologies Inc., Edmonton, Canada) or (ii) oral preparations of other root extracts of North American (*Panax quinquefolius*) or Asian ginseng (*Panax ginseng*). In trials using American or Asian ginseng root extracts other than COLD-fX, ginseng had to be the primary active ingredient. Appropriate comparators were placebo or no treatment. The studies identified in the search were initially screened for broad relevance by one reviewer (S. K.) based on their titles and abstracts. Subsequently, the full publications were retrieved and two reviewers (J. K. S. and S. K.) independently assessed the eligibility of potentially relevant trials using a standardized form. Disagreements were resolved by consensus.

### 2.3. Outcomes

The primary outcome was the incidence of common colds throughout the trial period. “Cold” was classified using the definition of the trial authors. Secondary outcomes included the severity, duration of colds, cold symptoms and adverse events. Trials that reported the combined incidence of any acute respiratory infections (ARIs) including common colds, such as the combined incidence of either cold or influenza, instead of measuring occurrences of common colds separately, were also included in the review.

### 2.4. Quality Assessment

Two reviewers (J. K. S. and S. K.) independently assessed the methodological quality of each included trial with the validated Jadad scale [4]. Concealment of treatment allocation was assessed using the criteria of Schulz and colleagues [5]. Disagreement between the reviewers regarding the quality ratings was resolved through discussion.

### 2.5. Data Extraction

Data were extracted independently by two reviewers (J. K. S. and S. K.). A standardized paper-based extraction form was used to collect details regarding the study design, population, intervention, measured outcomes and other relevant study characteristics (e.g. source of funding, language of publication, etc.). Agreement between the reviewers was confirmed prior to entering data.

### 2.6. Data Analysis

Analysis was conducted using Review Manager 4.2 [6]. Meta-analysis was performed to pool results across several trials where the population, intervention, comparison group and outcome were considered to be comparable. The *I* ^2^ statistic was used to assess all the pooled estimates for heterogeneity. Continuous outcomes were combined using a weighted mean difference, and the inverse variance method was used to assign weights to the trials. Relative risk for harm was used for the dichotomous outcome, and the Mantel-Haenszel method was used to assign weights to the trials [7].

For all meta-analyses, a random effects model was used. For each outcome measure, a point estimate and its respective 95% confidence interval (95% CI) were calculated. A forest plot was created for outcomes for which the results of two or more studies were pooled.

A subgroup analysis was planned *a priori* to compare trials using North American ginseng extracts to those using Asian ginseng extracts. Similarly, a sensitivity analysis was planned to compare the respective influence of studies of high and low quality. There was an insufficient number of trials to construct a funnel plot or to conduct a quantitative analysis to assess for publication bias.

## 3. Results

### 3.1. Study Selection

**Figure 1:**
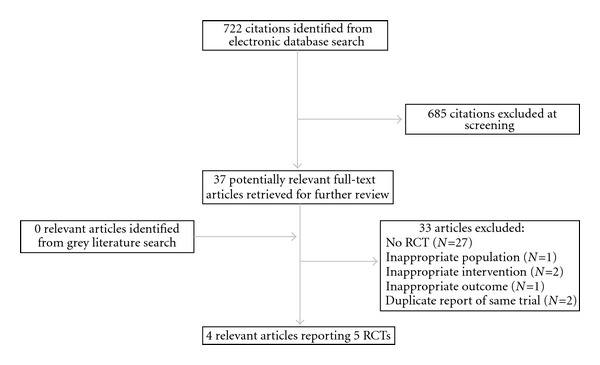
Flow diagram of study search and inclusion.

A flow diagram of the retrieval and selection process is shown in Figure 1. Independent review of the 37 potentially relevant studies identified five relevant RCTs published in four articles [8–11]. The paper by McElhaney and colleagues in 2004 [8] reported two separate parallel-arm RCTs conducted during two influenza seasons (8- and 16-week duration, resp.); the two trials are reported separately in this analysis (McElhaney 2004a and McElhaney 2004b). References of the excluded studies are available on request to the corresponding author.

A single ongoing study was identified through ClinicalTrials.gov. This trial examines the effectiveness of a single 800 mg dose of COLD-fX in preventing respiratory infections compared to a placebo in healthy adult employees of continuing care facilities. The expected completion date was April 2006. No additional information regarding this study could be retrieved.

### 3.2. Study Characteristics

The characteristics of the included trials are shown in Table 1. All of the five included trials were parallel-arm RCTs and were published in English between 1996 and 2006. Four of the RCTs [8, 10, 11] examined the efficacy of COLD-fX [a poly-furanosyl-pyranosyl-saccharide-rich extract of North American ginseng root (*Panax quinquefolius*)] in the prevention of colds compared to placebo, while the remaining trial [9] compared the prophylactic use of Ginsana G115 [a standardized extract of Asian ginseng root (*Panax ginseng*)] to placebo. All of the four COLD-fX trials were conducted in Canada and funded by the manufacturer, while the Ginsana trial was conducted in Italy and did not specify its source of funding. There was clinical heterogeneity among the five trials. Three trials included primarily elderly populations, with mean ages over 65 years [8, 11], while the remaining two trials examined middle-aged individuals [9, 10]. The settings included long-term care facilities in two [8] and communities in three trials [9–11], respectively.

Table 2 indicates the methodological quality ratings for each study on the components of the Jadad scale and on the allocation concealment criterion. Overall, methodological quality was quite variable: two studies had high-quality scores using both the Jadad criteria (rated as 5/5) and Schulz's allocation concealment criteria (rated adequate) [10, 11]. In contrast, the remaining three studies had much lower quality scores, ranking 2/5 on the Jadad scale and with unclear allocation concealment [8, 9]. The agreement between the reviewers in assessing quality was high (*κ* = 0.86).

### 3.3. Efficacy

Only one trial [10] reported outcomes specific to the common cold while the remaining four trials [8, 9, 11] reported combined data for ARIs including common colds. Therefore, both outcomes specific to common colds and those related to ARIs were combined in our analysis.

#### 3.3.1. Primary Outcome

**Figure 2:**
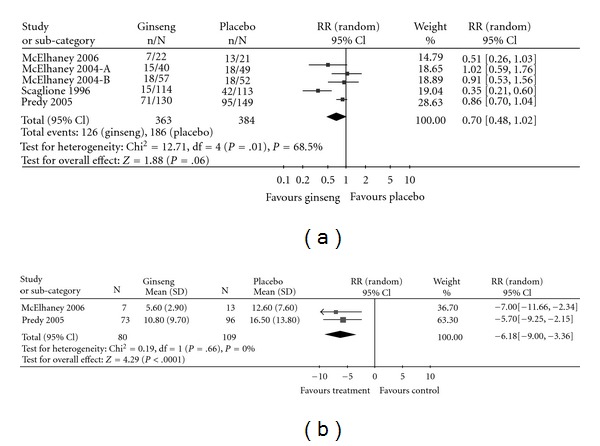
Forest-plot of incidence of having at least one cold or ARI (a) and duration of cold/ARI (b).

Five studies reported the proportion of participants who experienced at least one common cold or ARI throughout the duration of the study. The incidence of common colds and other ARIs are shown together in the Figure 2. The relative risk of acquiring at least one common cold or ARI was 0.70 (95% CI: 0.48–1.02) for study periods ranging from 8 to 16 weeks (Figure 2), with a large amount of heterogeneity (*I* ^2^ = 68.5%). The trials favored ginseng, with the exception of one [8], which showed no advantage of ginseng over placebo (relative risk 1.0). However, the size of the effect varied substantially across the trials and there was little overlap in the CIs between the studies.

The number of common colds throughout the study period was measured as a continuous outcome in one of the included trials [10]: Predy et al. found a statistically significant reduction of 25% (95% CI: 5–45%) in the total number of Jackson-verified colds experienced by the group taking COLD-fX compared to those taking the placebo. Jackson-verified colds were defined as a 2-day total symptom score greater than 14, where participants self-rated 10 cold symptoms on a scale ranging from 0 (no symptom) to 3 (severe symptom).

#### 3.3.2. Secondary Outcomes

Only one study [10] reported the effect of COLD-fX on the severity of cold symptoms. Daily, participants rated the severity of 10 cold symptoms (e.g. sore throat, runny nose, cough etc.) using a 4-point scale for each symptom, where a score of 0 indicated no symptom and 3 indicated severe symptom. The mean difference in the total symptom severity scores between the ginseng and placebo groups was −11.70 points over the 4-month study duration (95% CI: −33.69–10.29).

The duration of colds and ARIs was reported in two trials [10, 11]. Colds or ARIs in the ginseng group were an average of 6.2 days (95% CI: 3.4–9.0) shorter than in the placebo group (Figure 2). Heterogeneity between these two studies was negligible (*I* ^2^ = 0%).

Adverse events were assessed in all five trials. The overall incidence of adverse events reported varied widely from 4% [8] to 92% [8]. No trial found a statistically significant difference in the incidence of adverse events with the exception of Scaglione et al. [9] (7% in the ginseng group versus 1% in the placebo group, *P* = .04). The most common type of adverse event in all of the trials was “gastrointestinal symptoms”, reported in up to 45% of participants in one trial [8].

#### 3.3.3. Subgroup and Sensitivity Analyses

An *a priori* subgroup analysis was performed to examine whether the relative risk of acquiring a common cold differs between the four trials using COLD-fX [7, 9, 10], a standardized extract of North American ginseng, and the one study using Ginsana G115 [9], an Asian ginseng extract. The relative risk of cold or ARI using COLD-fX was 0.85 (95% CI: 0.72–1.01), while the relative risk using Ginsana G115 was 0.35 (95% CI: 0.21–0.60). Deeks' method indicated that the type of ginseng used (COLD-fX versus G115) was a significant factor for explaining heterogeneity between the trials (*Q* = 10.2, *P* = .001), where prophylactic use of Ginsana G115 was favored.

A sensitivity analysis for methodological quality indicated that high quality studies, scoring 5/5 on the Jadad scale [10, 11], had a relative risk of 0.74 (95% CI: 0.47–1.17) for acquiring a cold or ARI, while the remaining, low-quality studies (Jadad score of 2/5) had a relative risk of 0.69 (95% CI: 0.35–1.35) [8, 9]. Therefore, trials of high quality in this sample tended to have a more conservative estimate of treatment effect compared to trials of lower quality. The heterogeneity was much greater between the studies of low quality (*I* ^2^ = 78.8) and those of high quality (*I* ^2^ = 48.2). However, methodological quality was not significant in explaining heterogeneity (*Q* = 1.34, *P* = .247).

## 4. Discussion

The current review assessed the efficacy of preparations of North American and Asian ginseng extracts for the prevention of common colds. The incidence of common colds or ARIs in the ginseng group compared to placebo was assessed in five studies, yielding a nonsignificant 30% reduction favoring the ginseng group. However, methodological quality varied widely and there was little consistency in the size or precision of the effect. Pooling the results of two trials that assessed the duration of cold and ARI symptoms showed a statistically significant reduction by 6.2 days when comparing ginseng versus placebo.

The present review had set out to investigate the effect of ginseng extracts on preventing the common cold, yet the majority of the trials reported only prevention of ARIs including colds. Therefore, the results can only be applied to ARIs in general, including more severe illnesses such as influenza, rather than the common cold alone. In addition, there was limited data available to address the effect of ginseng on cold duration and symptom severity, and the methodological quality of the included studies varied substantially. All trials had restrictive eligibility criteria with regard to medical conditions, medications taken and other factors such as smoking and pregnancy. Therefore, the results of this review may only be reflective of healthy adults who do not have certain risk factors for colds and ARIs (e.g. smoking), and the effect may differ greatly in more vulnerable populations. In addition, a substantial proportion of the participants were elderly individuals, therefore caution should be used when generalizing the results of the pooled analyses to populations of younger adults. Finally, there was little consistency in the magnitude of the point estimate and minimal overlap of the confidence intervals across the trials. Considering the few number of trials and participants, the heterogeneous study populations, the varying methodological quality and the inconsistency of results between the trials, care must be taken in interpreting the efficacy of COLD-fX and G115 in cold prevention.

COLD-fX is marketed for long-term use for cold prevention as well as 3-day high-dose use when individuals perceive the onset of a cold. The currently available data are insufficient to support the use of COLD-fX for the prevention of common colds. The authors were unable to identify any past or current trials that examine the efficacy of short-term use of COLD-fX in the treatment of colds which have already begun. However, COLD-fX appears to be effective in reducing the duration of ARIs after onset, showing a mean decrease of 6 days in the length of colds/ARIs of the COLD-fX group compared to the placebo group. This result was statistically significant, however, it should be noted that this finding was based on only two trials whose populations were heterogeneous in terms of age. The heterogeneity of the populations and small sample size of one of the pooled studies may have contributed to the wide confidence interval of the estimate (Figure 2).

The efficacy of ginseng extracts must also be considered in light of potential adverse effects, which might occur as a result of its daily use. All studies reported adverse events and their frequency varied widely between the trials with the chief complaint being “gastrointestinal symptoms”. Two subjects in the ginseng group in the Predy et al. [10] trial developed type 2 diabetes during the study and were subsequently withdrawn. There was no information as to whether these adverse events were deemed to be related to ginseng. In the other four trials, no serious adverse events related to ginseng were reported. However, there is need for a systematic investigation of any harmful effects that might result from prolonged and frequent ingestion of these standardized ginseng extracts.

Further trials on the effect of ginseng extracts on the common cold and other ARIs are needed, especially on the short-term therapeutic use for which COLD-fX is advertised. The majority of the trials identified in this review did not use systematic criteria in defining the presence of common cold. Future trials should classify the occurrence of colds using a validated instrument, as this would likely decrease the potential for misclassification bias.

## 5. Conclusions

Currently, there is insufficient evidence to support the use of North American ginseng extracts in the prevention of common colds. While there was a trend toward a lower risk of developing at least one common cold in the ginseng group compared to the placebo group across the five trials, this result must be interpreted with caution due to inconsistency in the size of the effect and precision, as well as the varying quality of the included studies. There is some evidence consistent across two trials that the duration of colds and other ARIs is decreased by an average of 6 days for individuals taking the ginseng extract COLD-fX.

## Figures and Tables

**Table 1:** Characteristics of included studies.

Reference, country	Study design, duration, funding	Population (age, gender, setting)	Intervention (proprietary name, extract, dose)	Control (agent, dose)	Outcomes reported	Author's conclusions	Jadad score
McElhaney et al. [8], trial A, USA	RCT Parallel, 8 weeks, Manufacturer	82 years (mean), 20% male, Long-term care facility	*N* = 40, COLD-fX, North American ginseng, 2 × 200 mg day^−1^	*N* = 49, Placebo, Microcrystalline cellulose, 2 × 200 mg day^−1^	Clinically confirmed ARI, severity and duration of respiratory illness, laboratory-confirmed respiratory illness, severity and duration of influenza illness, AE.	COLD-fX was demonstrated to be potentially effective for the prevention of ARI, as well as safe and well tolerated	2/5
McElhaney et al. [8], trial B, USA	RCT Parallel, 12 weeks, Manufacturer	83.5 years (mean), 26% male, Long-term care facility	*N* = 57, COLD-fX, North American ginseng, 2 × 200 mg day^−1^	*N* = 52, Placebo, Microcrystalline cellulose, 2 × 200 mg day^−1^	Clinically confirmed ARI, severity and duration of respiratory illness, laboratory-confirmed respiratory illness, severity and duration of influenza illness, AE.	COLD-fX was demonstrated to be potentially effective for the prevention of ARI, as well as safe and well tolerated	2/5
McElhaney et al. [11], Canada	RCT Parallel, 16 weeks, Manufacturer	69 years (mean), 49% males, Community	*N* = 22, COLD-fX, North American ginseng, 2 × 200 mg day^−1^	*N* = 21, Placebo, Microcrystalline cellulose, 2 × 200 mg day^−1^	Incidence of ARI symptoms, duration of symptoms, AE.	COLD-fX reduced the relative risk and duration of respiratory symptoms, and is safe for daily use in immunocompetent seniors.	5/5
Predy et al. [10], Canada	RCT Parallel, 16 weeks, Manufacturer	43 years (mean), 40% males, Community	*N* = 130, COLD-fX, North American ginseng, 2 × 200 mg day^−1^	*N* = 149, Placebo, Rice powder, 2 capsules day^−1^	Incidence of colds reported and Jackson-verified, severity and duration of symptoms, AE.	Use of COLD-fX reduced the incidence of colds, proportion of subjects experiencing ≥2 colds, the severity and duration of colds.	5/5
Scaglione et al. [9], Italy	RCT Parallel, 12 weeks, Funding ND	48 years (mean), 58% males, Community, Multicenter	*N* = 114, Ginsana G115, *Panax ginseng*, 2 × 200 mg day^−1^	*N* = 113, Placebo, ND, 2 capsules day^−1^	Incidence of common colds and influenza, NK activity, specific antibody titres, AE.	Ginsana G115 helps improve human immune response and is able to protect against common cold and influenza.	2/5

AE: adverse events; ND: not described; NK: natural killer cells; RCT: randomized controlled trial.

**Table 2:** Methodological quality of included studies according to Jadad et al. [4] and Schultz et al. [5].

Reference	Described as randomized	Randomization well described and appropriate	Outcome assessment blinded	Blinding well described and appropriate	Description of withdrawals and drop-outs	Total Jadad score	Allocation concealment
McElhaney et al. [8], trial A	Yes	No	Yes	No	No	2/5	Unclear
McElhaney et al. [8], trial B	Yes	No	Yes	No	No	2/5	Unclear
McElhaney et al. [11]	Yes	Yes	Yes	Yes	Yes	5/5	Adequate
Predy et al. [10]	Yes	Yes	Yes	Yes	Yes	5/5	Adequate
Scaglione et al. [9]	Yes	No	Yes	No	No	2/5	Unclear

## Appendix

MEDLINE (Ovid) Search Strategy Version: OvidSP_UI1.0.1 1950 to November Week 2 2007 Adapted for controlled vocabulary of other electronic databases searched

1. exp Panax/
2. Plant Extracts/
3. Drugs, Chinese Herbal/
4. Plants, Medicinal/
5. Phytotherapy/
6. Plant Preparations/
7. Plant Roots/
8. Medicine, Herbal/
9. Polysaccharides/
10. (poly-furanosyl-pyranosyl-saccharide? or polyfuranosyl-pyranosyl saccharide? or polyfuranosyl-pyranosylsaccharide?).mp. [mp = title, original title, abstract, name of substance word, subject heading word]
11. (COLD-fX or Cold fX or ColdfX).mp. [mp = title, original title, abstract, name of substance word, subject heading word]
12. (CVT-E002 or CVT E002 or CVTE002).mp. [mp = title, original title, abstract, name of substance word, subject heading word]
13. (CNT-2000 or CNT 2000 or CNT2000).mp. [mp = title, original title, abstract, name of substance word, subject heading word]
14. (G-115 or G115).mp. [mp = title, original title, abstract, name of substance word, subject heading word]
15. ginseng$.mp.
16. panax.mp.
17. or/1–16
18. Common Cold/
19. Respiratory Tract Infections/
20. Respiratory Syncytial Virus, Human/
21. cold$.mp.
22. (common adj5 cold$).mp.
23. (respiratory adj5 (infection? or illness$)).mp.
24. URTI?.mp.
25. ARI?.mp.
26. or/18–25
27. 17 and 26
28. Animals/
29. Humans/
30. 28 not (28 and 29)
31. 27 not 30
